# Supplementary material for: Establishment and Characterization of a Spermatogonial Stem Cell Line from Tiger Puffer Fish (Takifugu rubripes)
Source: Animals (Basel). 2023 Sep 19;13(18):2959. doi: 10.3390/ani13182959 (PMC10525247; doi:10.3390/ani13182959)
Supplement: Supplementary file 1 [file animals-13-02959-s001.zip › animals-2366648-supplementary.pdf]

```
Takifugu_rubripes-_cox1_sequence.seq    TATAATCGGAGCCCCAGACATGGCCTTCCCCCGAATGAACAACATAAGCTTCTGACTGCTTCCCCCATCC    70
TrSSCs-cox1_sequence_.seq               TATAATCGGAGCCCCAGACATGGCCTTCCCCCGAATGAACAACATAAGCTTCTGACTGCTTCCCCCATCC    70
Consensus                               tataatcggagccccagacatggccttcccccgaatgaacaacataagcttctgactgcttcccccatcc

Takifugu_rubripes-_cox1_sequence.seq    TTCCTCCTTCTGCTCGCATCCTCTGGAGTAGAAGCCGGAGCGGGTACGGGCTGAACTGTTTACCCACCCC    140
TrSSCs-cox1_sequence_.seq               TTCCTCCTTCTGCTCGCATCCTCTGGAGTAGAAGCCGGAGCGGGTACGGGCTGAACTGTTTACCCACCCC    140
Consensus                               ttcctccttctgctcgcatcctctggagtagaagccggagcgggtacgggctgaactgtttacccacccc

Takifugu_rubripes-_cox1_sequence.seq    TAGCAGGAAATCTTGCCCACGCAGGGGCTTCTGTAGACCTCACCATCTTCTCTCTTCATCTTGCAGGGGT    210
TrSSCs-cox1_sequence_.seq               TAGCAGGAAATCTTGCCCACGCAGGGGCTTCTGTAGACCTCACCATCTTCTCTCTTCATCTTGCAGGGGT    210
Consensus                               tagcaggaaatcttgcccacgcaggggcttctgtagacctcaccatcttctctcttcatcttgcaggggt

Takifugu_rubripes-_cox1_sequence.seq    CTCCTCTATTCTAGGGGCAATCAACTTCATCACAACCATCATTAACATGAAGCCCCCAGCAATCTCACAA    280
TrSSCs-cox1_sequence_.seq               CTCCTCTATTCTAGGGGCAATCAACTTCATCACAACCATCATTAACATGAAGCCCCCAGCAATCTCACAA    280
Consensus                               ctcctctattctaggggcaatcaacttcatcacaaccatcattaacatgaagcccccagcaatctcacaa

Takifugu_rubripes-_cox1_sequence.seq    TACCAAACACCTCTTTTCGTGTGAGCCGTTTTAATTACTGCTGTACTTCTCCTGCTCTCCCTTCCAGTCC    350
TrSSCs-cox1_sequence_.seq               TACCAAACACCTCTTTTCGTGTGAGCCGTTTTAATTACTGCTGTACTTCTCCTGCTCTCCCTTCCAGTCC    350
Consensus                               taccaaacacctcttttcgtgtgagccgttttaattactgctgtacttctcctgctctcccttccagtcc

Takifugu_rubripes-_cox1_sequence.seq    TTGCAGCAGGGATTACAATACTTCTCACTGACCGAAACCTAAATACAACCTTCTTTGACCCAGCAGGAGG    420
TrSSCs-cox1_sequence_.seq               TTGCAGCAGGGATTACAATACTTCTCACTGACCGAAACCTAAATACAACCTTCTTTGACCCAGCAGGAGG    420
Consensus                               ttgcagcagggattacaatacttctcactgaccgaaacctaaatacaaccttctttgacccagcaggagg

Takifugu_rubripes-_cox1_sequence.seq    AGGAGACCCCATCTTGTACCAACACTTATTCTGATTCTTTGGACACCCTGAAGTCTACATTCTAATTCTC    490
TrSSCs-cox1_sequence_.seq               AGGAGACCCCATCTTGTACCAACACTTATTCTGATTCTTTGGACACCCTGAAGTCTACATTCTAATTCTC    490
Consensus                               aggagaccccatcttgtaccaacacttattctgattctttggacaccctgaagtctacattctaattctc

Takifugu_rubripes-_cox1_sequence.seq    CCTGGCTTCGGAATAATTTCACACATCGTAGCCTACTACTCGGGCAAAAAAGAACCATTCGGCTACATGG    560
TrSSCs-cox1_sequence_.seq               CCTGGCTTCGGAATAATTTCACACATCGTAGCCTACTACTCGGGCAAAAAAGAACCATTCGGCTACATGG    560
Consensus                               cctggcttcggaataatttcacacatcgtagcctactactcgggcaaaaaagaaccattcggctacatgg

Takifugu_rubripes-_cox1_sequence.seq    GCATGGTCTGAGCCATAATGGCCATCGGTCTTCTTGGTTTTATTGTATGAGCCCACCACATGTTTACAGT    630
TrSSCs-cox1_sequence_.seq               GCATGGTCTGAGCCATAATGGCCATCGGTCTTCTTGGTTTTATTGTATGAGCCCACCACATGTTTACAGT    630
Consensus                               gcatggtctgagccataatggccatcggtcttcttggttttattgtatgagcccaccacatgtttacagt

Takifugu_rubripes-_cox1_sequence.seq    CGGCATGGACGTAGACACCCGAGCCTACTTTACCTCTGCCACAATAATTATTGCCATCCCGACAGGAGTC    700
TrSSCs-cox1_sequence_.seq               CGGCATGGACGTAGACACCCGAGCCTACTTTACCTCTGCCACAATAATTATTGCCATCCCGACAGGAGTC    700
Consensus                               cggcatggacgtagacacccgagcctactttacctctgccacaataattattgccatcccgacaggagtc

Takifugu_rubripes-_cox1_sequence.seq    AAAGTATTTAGCTGACTTGCAACCTTGCATGGAGGATCAATTAAATGAGAAACCCCTATACTATGAGCCC    770
TrSSCs-cox1_sequence_.seq               AAAGTATTTAGCTGACTTGCAACCTTGCATGGAGGATCAATTAAATGAGAAACCCCTATACTATGAGCCC    770
Consensus                               aaagtatttagctgacttgcaaccttgcatggaggatcaattaaatgagaaacccctatactatgagccc

Takifugu_rubripes-_cox1_sequence.seq    TCGGCTTCATCTTCCTATTTACAGTGGGTGGCCTAACCGGAATTGTCCTAGCCAATTCATCCCTAGACAT    840
TrSSCs-cox1_sequence_.seq               TCGGCTTCATCTTCCTATTTACAGTGGGTGGCCTAACCGGAATTGTCCTAGCCAATTCATCCCTAGACAT    840
Consensus                               tcggcttcatcttcctatttacagtgggtggcctaaccggaattgtcctagccaattcatccctagacat

Takifugu_rubripes-_cox1_sequence.seq    CGTATTACACGACACCTACTACGTAGTTGCCCATTTCCACTAC                               883
TrSSCs-cox1_sequence_.seq               CGTATTACACGACACCTACTACGTAGTTGCCCATTTCCACTAC                               883
Consensus                               cgtattacacgacacctactacgtagttgcccatttccactac
```

Supplementary Figure S1. Sequence of *cox1* gene of *T. rubripes* and TrSSCs. The *cox1* PCR product was 883 bp in length. The sequence alignment analysis showed 100% similarity between the sequences from TrSSC and that from tiger puffer fish (Genbank accession number: AJ421455).
